# Acknowledgement to Reviewers of *Animals* in 2015

**DOI:** 10.3390/ani6010008

**Published:** 2016-01-21

**Authors:** Animals Editorial Office

**Affiliations:** MDPI AG, Klybeckstrasse 64, CH-4057 Basel, Switzerland; Animals@mdpi.com

The editors of *Animals* would like to express their sincere gratitude to the following reviewers for assessing manuscripts in 2015. 

We greatly appreciate the contribution of expert reviewers, which is crucial to the journal’s editorial decision-making process. Several steps have been taken in 2015 to thank and acknowledge reviewers. Good, timely reviews are rewarded with a discount off their next MDPI publication. By creating an account on the submission system, reviewers can access details of their past reviews, see the comments of other reviewers, and download a letter of acknowledgement for their records. In addition, MDPI has launched a collaboration with Publons, a website that seeks to publicly acknowledge reviewers on a per journal basis. This is all done, of course, within the constraints of reviewer confidentiality. Feedback from reviewers shows that most see their task as a voluntary and mostly unseen work in service to the scientific community. We are grateful to our reviewers for the contribution they make.

Abuelo Sebio, Ángel
Adamson, Dr Seana
Adler, Steffen A.
Aguayo-Ulloa, Lorena A.
Alberghina, Daniela
Arakawa, Takamitsu
Asher, Lucy
Azarpajouh, Samaneh
Baciadonna, Luigi
Bacon, Heather J.
Baker, Sandra
Banyard, Ashley
Bao, Jun
Barnard, Shanis
Bartošová, Jitka
Baumans, Vera
Beausoleill, Ngaio
Beck, Alan
Beltman, Marijke
Bennett, David
Berard, Joel
Berliner, Elizabeth
Bermingham, Emma N.
Biagi, P.
Bich, Leonardo
Biscarini, Filippo
Bobe, Gerd
Bonos, Eleftherios
Bouwman, Aniek C
Briefer, Elodie
Brook, Ryan K.
Brown, Wendy
Brunetti-Pierri, Nicola
Bucci, Marco
Buchner, Heinz Hans Florian
Butterworth, Andrew
Camerlink, Irene
Campbell, Madeleine
Carmichael, Samuel P.
Cartner, Samuel Corbin
Casey, Theresa M.
Chandler, Marjorie
Chang, Yung-fu
Chapwanya, Aspinas
Charmley, Ed
Clark, Dan
Clouard, Caroline
Cools, An
Coombe, Jo
Corbee, Ronald Jan
Coulson, Graeme
Coulter, Kendra
Crosson, Paul
Daigle, Courtney
De Brauwere, Nicolas
de Godoy, Maria
DeAraugo, Jodi
Demmers, Theo
Dixon, Laura
Douglas, Catherine
Dumbell, Lucy
Duziński, Kamil
Eckery, Douglas C.
Edgar, Joanne
Eicher, Susan
Elvidge, Chris
Estienne, Mark
Farmer-Dougan, Valeri
Farnworth, Mark
Farnworth, Mark J.
Fei, Andrew Chang-Young
Félix, Ananda Portela
Ferrari, Arianna
Ferraz, Guilherme de Camargo
Feuerbacher, Erica
Fidani, Cristiano
Fratkin, Jamie L.
Fredeen, Alan
Freire, João Pedro Bengala
Freire, Raf
Fuente, Jesús de la
Gabbi, Alexandre
Garcia, Arlene
Gard, Julie A.
Gazzano, Angelo
Geers, Rony
Geiger, Martha
German, Alexander J.
Giannenas, Ilias
Gillett, James
Godfrey, Robert W.
Goerlich-Jansson, Vivian
Goodman-Milne, Emma
Gourkow, Nadine
Grandin, Temple
Grinsted, Lena
Gruber, Leonhard
Gruber, Kenneth J.
Hart, Benjamin
Hasler, Rebecca M
Hassouna, Mélynda
Hawkins, Penny
Hawson, Lesley A.
Hazel, Susan
Heath, Cheryl
Heath, David
Heerkens, Jasper
Heiderstadt, Kathleen
Hemsworth, Lauren M.
Henry, Beverley
Hickman, Debra
Hitchens, Peta
Hoenig, Margarethe
Hoffman, Christy L.
Hofmeister, Erik K.
Hogg, Rachel
Horn, Marco
Hornick, Jean-Luc
Hosey, Geoff
Howard, Veronica
Howell, Gwyneth
Janczak, Andrew Michael
Ji, Weihong
Johnson, Sally
Jongman, Ellen C.
Jorgensen, Grete
Kendall, Holli A.
Kinsley, Marc
Kirkpatrick, Jay
Knappe-Poindecker, Maren
Knight, Peter
Koch, Katrin
Koppel, Kadri
Kougioumtzis, Alexandros
Krawczel, Peter
Kroll, Tina
Kuhla, Blörn
Kuhne, Franziska
laer, Eva Van
Lambert, Kim
Lambooij, Bert
Larsen, Jennifer
Laven, Richard
Leroy, Jo
Leuenberger, Fanny
Leytem, April
Li, Robert W.
Lima, Eduardo M. M.
Lord, Linda
Macciotta, Nicolò Pietro Paolo
Main, David
Margalida, Antoni
Marzano, Mariella
Mason, Georgia J.
Masterton, Malin
Mastilović, Jasna S.
Maurice van Eijndhoven, M.H.T
Mawson, Peter R.
McCobb, Emily
McGee, Mark
Miguel-Pacheco, Giuliana G.
Minero, Michela
Moons, Christel P. H.
Morgan, Karin
Morton, David
Moyes, Kasey M.
Muñoz-García, Claudia I.
Musiani, Marco
Negro Balmaseda, Juan José
Neumann, Inga D.
Niehaus, Andrew
Noble, Glenys
O’dwyer, Lisel A.
Oberbauer, Anita M.
Ohl, Frauke
Ohtani, Nobuyo
Opsomer, Geert
Ormandy, Elisabeth H.
Palmer, Andrew J.
Pardon, Bart
Passantino, Annamaria
Pastorelli, Grazia
Pechová, Alena
Peham, Christian
Perkins, Nathan
Petersen, Søren O.
Petitclerc, Denis
Place, Sara E.
Plummer, Paul
Porto, Simona M. C.
Prescott, Mark
Protopopova, Alexandra
Proudfoot, Katy
Rasmussen, Morten Dam
Reecy, James
Reed, Brian
Reimert, Inonge
Riber, Anja Brinch
Riley, Chris
Robins, Andrew
Rocca, Giorgia Della
Rodenburg, T. Bas
Rodger, Jennifer
Roger, Paul
Rogers, Chris W.
Rollin, Bernard
Roth, Lina S.V.
Ruviaro, Clandio
Sakomura, Nilva Kazue
Sampaio, Cristiano
Sanchez, Nicole
Sargisson, Rebecca J.
Scheelings, Titus Franciscus
Schiavone, Achille
Schlageter-Tello, Andrés
Schuller, Simone
Schuppli, Catherine A.
Schutz, Karin
Scott, Marian
Sheldon, Martin
Shields, Sara
Siegford, Janice M.
Singh, Bhupinder
Slater, Margaret
Slater, Margaret R.
Smith, Martin
Snowden, Karen
Speijers, Marijntje H. M.
Spielman, Derek
Stratmann, Ariane
Takeshi, Tsuka
Talling, Janet
Tanaka, Tomomi
Taylor, Andy
Taylor, Mel
Teixeira, Dayane Lemos
Tilbrook, Alan
Turner, Patricia V.
Turner, Dennis
Van Herzele, Ann
Vogel, Kurt
Wakshlag, Joseph
Walker, Jessica K.
Warburton, Bruce
Warren-Smith, Amanda
Weary, Daniel M.
Webb, Jim
Weeth, Lisa P.
Weng, Hsin-Yi
Weston, Michael A.
White, Sara C.
Whiting, Martin
Wichert, Brigitta
Williams, J.M.
Willis, Craig K.R.
Wolframm, Inga A.
Yoak, Andrew J.
Yun, Jinhyeon
Zaninelli, Mauro
Zebeli, Qendrim
Zhang, Zhigang
Zurlo, Joanne

